# Bootstrap vs asymptotic variance estimation when using propensity score weighting with continuous and binary outcomes

**DOI:** 10.1002/sim.9519

**Published:** 2022-07-15

**Authors:** Peter C. Austin

**Affiliations:** ^1^ ICES Toronto Ontario Canada; ^2^ Institute of Health Policy Management and Evaluation, University of Toronto Toronto Ontario Canada; ^3^ Sunnybrook Research Institute Toronto Ontario Canada

**Keywords:** bootstrap, inverse probability of treatment weighting, propensity score, variance estimation

## Abstract

We used Monte Carlo simulations to compare the performance of asymptotic variance estimators to that of the bootstrap when estimating standard errors of differences in means, risk differences, and relative risks using propensity score weighting. We considered four different sets of weights: conventional inverse probability of treatment weights with the average treatment effect (ATE) as the target estimand, weights for estimating the average treatment effect in the treated (ATT), matching weights, and overlap weights. We considered sample sizes ranging from 250 to 10 000 and allowed the prevalence of treatment to range from 0.1 to 0.9. We found that, when using ATE weights and sample sizes were ≤ 1000, then the use of the bootstrap resulted in estimates of SE that were more accurate than the asymptotic estimates. A similar finding was observed when using ATT weights and sample sizes were ≤ 1000 and the prevalence of treatment was moderate to high. When using matching weights and overlap weights, both the asymptotic estimator and the bootstrap resulted in accurate estimates of SE across all sample sizes and prevalences of treatment. Even when using the bootstrap with ATE weights, empirical coverage rates of confidence intervals were suboptimal when sample sizes were low to moderate and the prevalence of treatment was either very low or very high. A similar finding was observed when using the bootstrap with ATT weights when sample sizes were low to moderate and the prevalence of treatment was very high.

## 1 INTRODUCTION

Propensity score weighting is a popular causal inference method for estimating the effects of treatments, exposures, and interventions when using observational data. The propensity score is the probability of a subject being treated (vs receiving the control) based on the subject's observed baseline covariates.^1^ Propensity score weighting entails constructing weights that are derived from the propensity score. The first set of propensity‐score based weights, referred to as inverse probability of treatment weights (IPTW), weight each subject by the inverse of the probability of receiving the treatment that subject received.^2^ Just as randomization will, in expectation, balance baseline covariates between treated and control subjects, similarly, incorporating these weights will, in expectation balance measured baseline covariates between treated and control subjects. The use of IPTW allows one to estimate the average treatment effect (ATE).

Since the introduction of IPT weights, other sets of weights have been proposed. These include weights that allow one to estimate the average treatment effect in the treated (ATT), matching weights (MW), and overlap weights (OW).^3^, ^4^, ^5^, ^6^, ^7^ The latter two sets of weights target inference at those subjects for whom there is the greatest equipoise in treatment selection.

Asymptotic standard errors have been described for use with these different sets of weights.^4^, ^5^, ^8^, ^9^ These estimated standard errors permit construction of confidence intervals for estimated treatment effects along with tests of statistical significance of the estimated treatment effects. The bootstrap is a commonly‐used resampling methods for estimating SEs.^10^ The objective of the current study was to compare the use of the bootstrap with the use of asymptotic variance estimators when estimating the SE of estimated difference in means, risk differences, and relative risks. We consider scenarios with small, moderate, and large sample sizes, as well as low, medium, and high prevalences of treatment.

## 2 MONTE CARLO SIMULATIONS–METHODS

We conducted a series of Monte Carlo simulations to compare the performance of asymptotic variance estimators with the use of the bootstrap when estimating differences in means, risk differences, and relative risks when using propensity score‐based weights. We examined four different sets of weights.

### 2.1 Factors in the Monte Carlo simulations

We allowed two factors to vary in the Monte Carlo simulations: the size of the random sample drawn from the super‐population and the prevalence of treatment in the super‐population. The former took on 5 values: 250, 500, 1000, 5000, and 10 000. The latter took on nine values: from 0.1 to 0.9 in increments of 0.1. We used a full factorial design and thus considered 45 different scenarios. For the purposes of the current study, we made the admittedly subjective classification that sample sizes of 250 and 500 are small, sample sizes of 1000 are moderate, and sample sizes of 5000 or 10 000 are large.

### 2.2 Data‐generating process

For a given scenario we simulated subjects from a super‐population of size 1 000 000. For each subject we generated 10 baseline covariates from a multivariate normal distribution with mean zero, such that the variance of each of the 10 covariates was equal to 1 and the correlation between any pair of distinct covariates was 0.2. The first five baseline covariates ($x_{1},\ldots ,x_{5}$) were retained as continuous covariates. The second set of baseline covariates ($x_{6},\ldots ,x_{10}$) were dichotomized at the 10th, 20th, 30th, 40th, and 50th percentiles, respectively, so that the prevalence of the five binary covariates were 10%, 20%, 30%, 40%, and 50%, respectively.

We then generated a binary treatment variable for each subject using the following logistic model: $\begin{array}{l} \text{logit}\left(p_{i,\text{treat}}\right)=\alpha_{0,\text{treat}}+\log\left(1.1\right)x_{1,i}+\log\left(1.2\right)x_{2,i}+\log\left(1.5\right)x_{3,i}+\log\left(1.75\right)x_{4,i}+\log\left(2\right)x_{5,i}+\\ \;\log\left(1.25\right)x_{6,i}+\log\left(1.5\right)x_{7,i}+\log\left(2\right)x_{8,i}+\log\left(0.8\right)x_{8,i}+\log\left(0.5\right)x_{10,i}. \end{array}$

Thus, each of the 10 baseline covariates was associated with receipt of treatment. For each subject, a binary treatment variable was generated from a Bernoulli distribution with subject‐specific parameter equal to $p_{i,\text{treat}}$: $Z_{i}∼\mathrm{Be}\left(p_{i,\text{treat}}\right)$ (*Z* = 0 for control; *Z* = 1 for treated). The intercept of the treatment‐selection model ($\alpha_{0,\text{treat}}$) was determined using a bisection approach so that the prevalence of treatment in the super‐population was the desired quantity. The value of $\alpha_{0,\text{treat}}$ was −2.69, −1.69, −1.00, −0.42, 0.13, 0.67, 1.25, 1.95, and 2.93 for prevalences of treatment from 0.1 to 0.9, respectively.

The degree of overlap in the distribution of the propensity score between treated and control subjects is described in Figure A1 in the online supplemental material. There is one panel for each of the nine prevalences of treatment. In all scenarios, the c‐statistic of the propensity score model was approximately 0.80.

We then generated both a continuous outcome and a binary outcome for each subject. Continuous outcomes were simulated using the following linear model:

(1) $$Y_{i}=2Z_{i}+2.5x_{1,i}+2x_{2,i}+1.5x_{3,i}+1x_{4,i}+0.5x_{5,i}+2x_{6,i}+1x_{7,i}+5x_{8,i}+4x_{8,i}+3x_{10,i}+\varepsilon_{i},$$

$\text{where}\;\varepsilon_{i}∼N\left(0,\sigma^{2}\right).$ Thus, each of the 10 baseline covariates was associated with the continuous outcome. The variance of the error distribution ($\sigma^{2}$) was chosen so that variation in the 10 baseline covariates explained 25% of the variation of the outcome in untreated subjects. Under this data‐generating process, treatment increased the mean outcome by 2 units. Since there was a uniform treatment effect, both the ATE and the ATT were equal to 2. Similarly, the true target estimands when using matching weights and overlap weights (see below for definitions) were also 2.

Binary outcomes were generated using the following logistic model:

(2) $$\begin{array}{ll} & \text{logit}\left(p_{i,\text{outcome}}\right)=\alpha_{0,\text{outcome}}+\alpha_{\text{treat}}Z_{i}+\log\left(2\right)x_{1,i}+\log\left(1.75\right)x_{2,i}+\log\left(1.1\right)x_{3,i}+\log\left(1.5\right)x_{4,i}+\\ & \log\left(1.2\right)x_{5,i}+\log\left(2\right)x_{6,i}+\log\left(1.5\right)x_{7,i}+\log\left(1.1\right)x_{8,i}+\log\left(1.25\right)x_{8,i}+\log\left(2\right)x_{10,i}. \end{array}$$

We first used a bisection procedure to determine the intercept of the outcome model $\left(\alpha_{0,\text{outcome}}\right)$ so that the prevalence of the outcome in the super‐population was 0.2 if all subjects were untreated. Having determined the intercept, we then used a second bisection procedure to determine the log‐odds ratio for treatment $\left(\alpha_{\text{treat}}\right)$ so that the risk difference for treatment was −0.02 with the ATE as the target estimand. This was done by generating outcomes under treatment and control for each subject (these are the two potential outcomes). Let $Y_{i}\left(0\right)$ and $Y_{i}\left(1\right)$ denote the two potential outcomes for the *i*th subject (the outcomes under control and treatment, respectively). In the simulated super‐population, we determined the mean of $Y_{i}\left(1\right)-Y_{i}\left(0\right)$ across the super‐population. We modified the value of $\alpha_{\text{treat}}$ until the mean was −0.02. Thus, the ATE risk difference was −0.02. To determine the true ATT risk difference, we determined the mean of $Y_{i}\left(1\right)-Y_{i}\left(0\right)$ across all *treated* subjects in the super‐population (rather than across all subjects). In order to determine the true value of the ATE relative risk, we determined $\frac{\frac{1}{1\;000\;000}\sum_{i=1}^{1\;000\;000}Y_{i}\left(1\right)}{\frac{1}{1\;000\;000}\sum_{i=1}^{1\;000\;000}Y_{i}\left(0\right)}$. To determine the true value of the ATT relative risk, the two averages in the previous sentence were taken over all treated subjects in the super‐population, rather than over the entire super‐population. We determined the true target estimand when using matching weights and overlap weights by determining the weighed proportion of the outcome in treated and control subjects separately using the true known values of the given weight and then computed the difference (risk difference) and ratio (relative risk) of these weighted proportions.

### 2.3 Statistical analyzes

We drew a random sample of size N from the super‐population. In the random sample we estimated the propensity score using a logistic regression model in which the binary treatment status variable was regressed on the 10 baseline covariates. We then estimated four different sets of weights: ATE weights, ATT weights, MW, and OW.^2^, ^3^, ^4^, ^5^, ^6^ Let e(X) denote the estimated propensity score for a subject with covariate vector X and let *Z* denote treatment status (*Z* = 0 for control; *Z* = 1 for treated). These four sets of weights are defined as:

$$w_{\mathrm{ATE}}\left(X\right)=\frac{Z}{e\left(X\right)}+\frac{1-Z}{1-e\left(X\right)},$$

$$w_{\mathrm{ATT}}\left(X\right)=Z+\frac{\left(1-Z\right)e\left(X\right)}{1-e\left(X\right)},$$

$w_{\mathrm{MW}}\left(X\right)=\frac{Z\min\left(e\left(X\right),1-e\left(X\right)\right)}{e\left(X\right),}+\frac{\left(1-Z\right)\min\left(e\left(X\right),1-e\left(X\right)\right)}{1-e\left(X\right),},$ and $w_{\mathrm{OW}}\left(X\right)=Z\left(1-e\left(X\right)\right)+\left(1-Z\right)\left(e\left(X\right)\right).$

The use of the first set of weights targets the ATE, while the second set of weights targets the ATT. The latter two sets of weights target inference at the subpopulation for whom there is the greatest clinical equipoise about treatment.^4^

In the random sample of size *N*, we estimated the absolute weighted standardized mean difference for each of the 10 baseline covariates,^11^, ^12^ and determined the maximum absolute weighted standardized mean difference across the 10 covariates. We also computed the effective sample size of the weighted sample.^13^

In the random sample of size *N*, we estimated the difference in means (continuous outcome), the risk difference (binary outcome) and the logarithm of the relative risk (binary outcome) using each of the four sets of weights (we estimated the logarithm of the relative risk as its sampling distribution is more likely to be normal compared to the sampling distribution of the relative risks). The difference in means and the risk difference were estimated as $\frac{\sum_{i=1}^{N}w_{1}\left(X\right)Z_{i}Y_{i}}{\sum_{i=1}^{N}w_{1}\left(X\right)Z_{i}}-\frac{\sum_{i=1}^{N}w_{0}\left(X\right)\left(1-Z_{i}\right)Y_{i}}{\sum_{i=1}^{N}w_{0}\left(X\right)\left(1-Z_{i}\right)}$, where $w_{1}\left(X\right)\;\text{and}\;w_{0}\left(X\right)$ are the weights under treatment and control, respectively, and where *Y* denotes the continuous or binary outcome. The relative risk was estimated as $\frac{\sum_{i=1}^{N}w_{1}\left(X\right)Z_{i}Y_{i}}{\sum_{i=1}^{N}w_{1}\left(X\right)Z_{i}}/\frac{\sum_{i=1}^{N}w_{0}\left(X\right)\left(1-Z_{i}\right)Y_{i}}{\sum_{i=1}^{N}w_{0}\left(X\right)\left(1-Z_{i}\right)}$.

Standard errors of the differences in means, risk difference, and the logarithm of the relative risk were obtained using the asymptotic variance estimators described elsewhere.^4^, ^5^, ^8^, ^9^ We constructed 95% confidence using standard normal‐theory methods based on the estimated SE of the estimated treatment effect. We then determined whether the estimated 95% confidence interval contained the true value of the treatment effect from the data‐generating process.

We then used the bootstrap to estimate SEs of the estimated treatment effects.^10^ To do so, we drew 200 bootstrap samples from the random sample. In each of these bootstrap samples, the propensity score was re‐estimated and each set of weights was computed using the newly estimated propensity scores. The difference in means, risk difference, and the logarithm of the relative risk was then computed in each bootstrap sample using each of the four sets of weights. The bootstrap estimate of the SE of a given estimate in the original sample (eg, the difference in means) was equal to the SD of the corresponding quantity across the 200 bootstrap samples.

The above set of analyzes was repeated 1000 times. We thus drew 1000 samples, and in each sample we obtained an estimate of the difference in means, the risk difference, and the logarithm of the relative risk using each set of weights. We also obtained an asymptotic estimate of the SE of each estimate along with a bootstrap estimate of the SE of each estimate. For each estimand (difference in mean, risk difference, and relative risk) and for each set of weights (ATE, ATT, MW, and OW), we compared the mean estimated SE (both the asymptotic estimate and the bootstrap estimate) across the 1000 simulation replicates with the SD of the estimated treatment effect across the 1000 simulation replicates. The ratio is defined as $\frac{\frac{1}{1,000}\sum_{i=1}^{1,000}\mathrm{se}\left(\varphi_{i}\right)}{\mathrm{SD}\left(\varphi_{i}\right)}$, where $\varphi_{i}$ denotes the estimated treatment effect (difference in means, risk difference, logarithm of the relative risk) in the *i*th simulation replicate, $\mathrm{se}\left(\varphi_{i}\right)$ denotes the estimated SE in the *i*th simulation replicate and $\mathrm{SD}\left(\varphi_{i}\right)$ denotes the SD of the estimated treatment effect across the 1000 simulation replicates. If this ratio is equal to one, then the estimated SE is correctly estimating the SD of the sampling distribution of the estimated treatment. We also computed the proportion of confidence intervals that contained the true value of the treatment effect. We also computed the mean of the maximum absolute standardized mean difference and the mean effective sample size across the 1000 simulation replicates.

### 2.4 Software

The simulations were conducted using the R statistical programming language (version 3.6.3). The weighted analyzes were conducted using the PSweight function from the PSweight package. Within the PSweight function, we estimated the propensity score using logistic regression in which treatment status was regressed on the measured baseline covariates. In PSweight, the variance of the estimated treatment effect is obtained using the empirical sandwich variance for propensity score weighting estimators based on M‐estimation theory.^14^ This variance estimator accounts for the uncertainty in estimating the propensity score. The reader is referred elsewhere for further details.^14^

## 3 MONTE CARLO SIMULATIONS–RESULTS

### 3.1 Covariate balance and effective sample size

**FIGURE 1:**
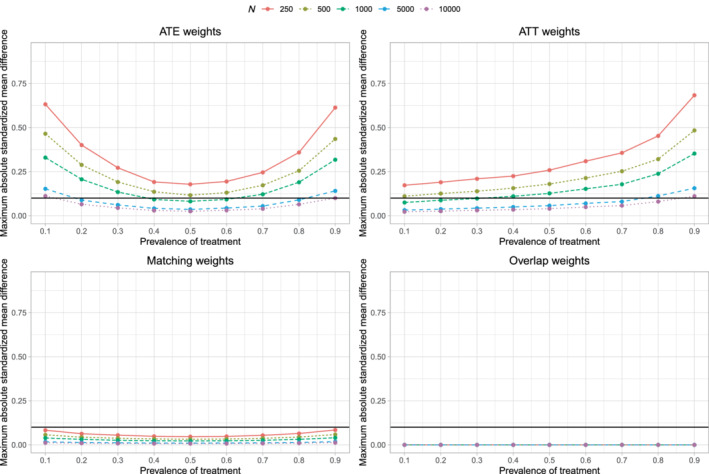
Covariate balance after weighting

The maximum mean standardized difference across the simulation replicates is reported in Figure 1. There is one panel for each of the four sets of weights. On each panel we have superimposed a horizontal line denoting a standardized difference of 0.1, which some have used to denote acceptable balance.^15^ When using ATE, ATT, and matching weights, one observes that, for a given prevalence of treatment, balance improved with increasing sample size, reflecting that balance is a large sample property. When using ATE weights, for a given sample size, the best balance was observed when the prevalence of treatment was 0.5. When using ATT weights, for a given sample size, the best balance was observed when the prevalence of treatment was 0.1. When using matching weights, for a given sample size, the best balance was observed when the prevalence of treatment was 0.5; however, prevalence of treatment and sample size had a much weaker effect on balance for matching weights than for ATE and ATT weights. When using overlap weights, the maximum standardized difference was 0 across all scenarios, as would be expected.^13^

The mean effective sample sizes across the 45 different scenarios are reported in Figure A2 in the online supplemental material. When using ATE weights, for a given sample size, the effective sample size was lowest when the prevalence of treatment was 0.5. When using ATT weights, for a given sample size, the effective sample size was lowest when the prevalence of treatment was 0.1. When using matching weights or overlap weights, for a given sample size, the effective sample size was largest when the prevalence of treatment was 0.5. The latter observation is as anticipated, as these latter two sets of weights place greater weight on subjects for whom there is greater equipoise (ie, for whom the propensity score is close to 0.5).

### 3.2 Comparison of estimated SE to the SD of the sampling distribution

**FIGURE 2:**
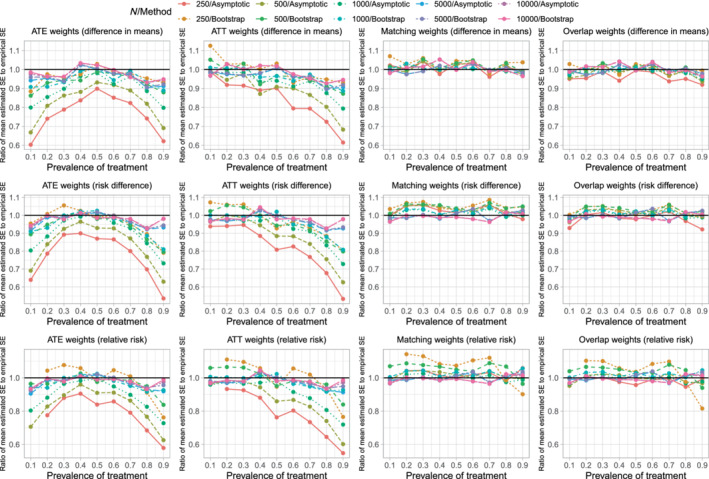
Ratio of mean estimated SE to empirical SE

The ratios of the mean estimated SEs to the standard deviations of the estimated treatment effect are reported in Figure 2. There are 12 panels, one for each combination of the three estimands and the four sets of weights. Each panel consists of 10 lines, one line for each combination of sample size and variance estimation method (asymptotic vs bootstrap). The panels for a given estimand use the same vertical axis scale so that results can be easily compared across the different sets of weights.

When using ATE weights, variance estimation tended to be most accurate, regardless of the variance estimation method, when the prevalence of treatment was 0.5. The accuracy of variance estimation tended to decrease as the prevalence of treatment decreased toward 0.1 or increased toward 0.9. As expected, when using the asymptotic variance estimator, the accuracy of the variance estimate increased with increasing sample size. When the sample size was small (250 or 500) or moderate (1000), the use of the bootstrap tended to result in more accurate estimates of SE than did the use of the asymptotic estimator. When sample size was large (5000 or 10 000) the asymptotic estimator had approximately the same accuracy as the bootstrap estimator. Except when both the sample size was large and the prevalence of treatment was close to 0.5, the ratio of mean estimated SE to the empirical SE tended to less than one, indicating that the estimated SE tended to underestimate the SD of the sampling distribution of the treatment effect.

When using ATT weights, the variance estimate tended to be most accurate when the prevalence of treatment was very low (0.1) and accuracy decreased as the prevalence of treatment increased. As with the ATE weights, when sample size was small or moderate, the use of the bootstrap tended to result in more accurate estimates of SE than did the use of the asymptotic estimator. When sample size was large, the asymptotic estimate had approximately the same accuracy as the bootstrap estimator.

When using matching weights or overlap weights, the accuracy of the variance estimator displayed little variability across estimation methods, sample size, and prevalence of treatment.

The above observations were consistent, regardless of whether one was estimating difference in means (continuous outcomes), risk differences (binary outcomes), or relative risk (binary outcomes).

### 3.3 Empirical coverage rates of estimated 95% confidence intervals

**FIGURE 3:**
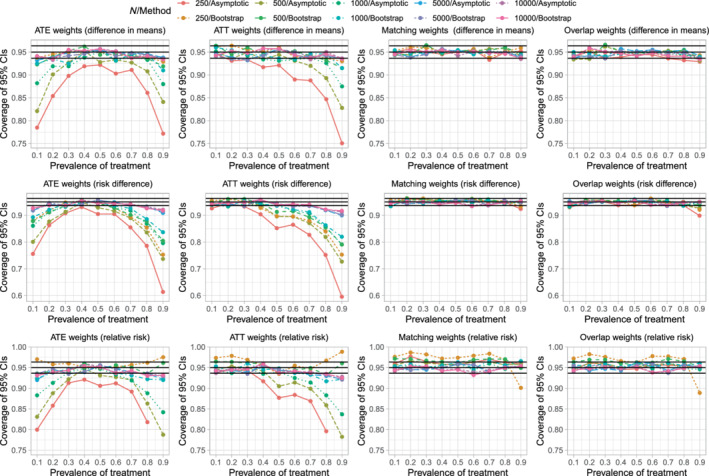
Empirical coverage rates of 95% confidence intervals

The empirical coverages rates of estimated 95% confidence intervals are reported in Figure 3, which has a similar structure to that of Figure 2. On each panel we have superimposed three horizontal lines denoting coverage rates of 0.95, 0.9365, and 0.9635. The first denotes the advertised coverage rate. The latter two denote thresholds, such that empirical coverage rates that are less than 0.9365 or greater than 0.9635 are statistically significantly different from the advertised rate of 0.95 using a 5% significance level, based on a standard normal‐theory test and our use of 1000 simulation replicates.

When using ATE weights, the empirical coverage rates tended to display an inverted U‐shaped relationship with the prevalence of treatment, with empirical coverage rates tending to achieve a maximum when the prevalence of treatment was 0.5. When the prevalence of treatment was either very low or very high and the sample size was low to moderate, then empirical coverage rates tended to be statistically significantly lower than advertised. When sample sizes were low to moderate and the prevalence of treatment was either very low or very high, the use of the bootstrap tended to result in confidence intervals whose empirical coverage rates were better than those obtained using the asymptotic variance estimator. However, even with the use of the bootstrap, coverage rates tended to be suboptimal when the sample size was low to moderate and prevalence of treatment was either very low or very high.

When using ATT weights, empirical coverage rates tended to be as advertised, except when the prevalence of treatment was very high and the sample size was low to moderate. When the prevalence of treatment was 0.9 and the sample size was low or moderate, the use of the bootstrap improved empirical coverage rates, but the empirical coverage rates remained suboptimal.

When using matching weights or overlap weights, empirical coverage rates tended to be not statistically significantly different from the advertised rate.

The above observations tended to be consistent, regardless of whether one was estimating difference in means (continuous outcomes), risk differences (binary outcomes), or relative risk (binary outcomes).

When using ATE weights, the low coverage rates of 95% confidence intervals that were observed when the prevalence of treatment was either very low or very high was not due to the effective sample size. In Figure A2, one notes that, with ATE weights, the effective sample size was highest when the prevalence of treatment was either very low or very high. Similarly, when using ATT weights, the low coverage rates that were observed when the prevalence of treatment was very high was not due to the effective sample size, as effective sample size was highest when the prevalence of treatment was very high.

We hypothesize that the suboptimal coverage rates of estimated 95% confidence intervals using ATE or ATT weights is due to residual imbalance in measured baseline covariates. The scenarios in which coverage rates were suboptimal (Figure 3) are the same as those scenarios in which there was residual imbalance in baseline covariates (Figure 1).

## 4 SENSITIVITY ANALYZES: TREATMENT‐COVARIATE INTERACTIONS IN THE OUTCOME MODEL

In the extensive set of simulations described above, the effect of treatment (on either the means scale (continuous outcome) or the odds ratio scale (binary outcome)) was constant across covariate patterns. We conducted a sensitivity analysis in which this assumption was relaxed. This set of simulations was identical to those described above with one exception.

### 4.1 Methods

The regression models for simulating outcomes (formula (1) for continuous outcomes and formula (2) for binary outcomes) were modified to include interactions between treatment status (*Z*) and each of the 10 baseline covariates. In particular, they modified to include $\frac{1}{4}\beta_{j}Z\times X_{j}$, for *j* = 1,…,10, and where $\beta_{j}$ is the main effect for $X_{j}$ (ie, for each covariate, the coefficient for the interaction term is 0.25 times the coefficient for the main effect).

### 4.2 Results

**FIGURE 4:**
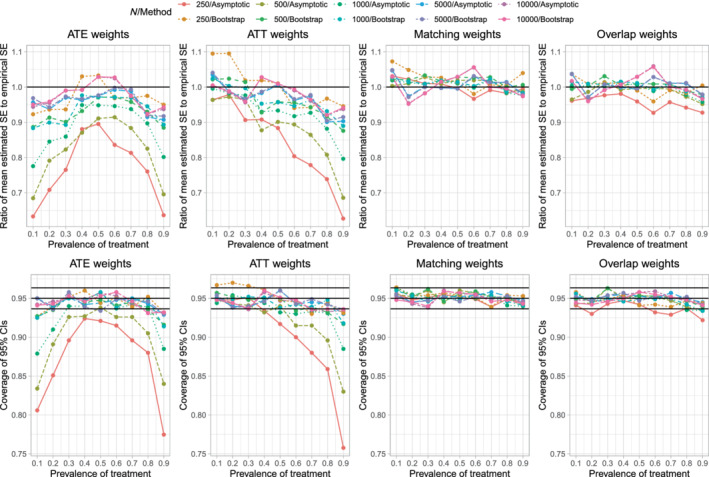
Sensitivity analysis–interactions (Estimand: difference in means)

We report results for estimating differences in means for the continuous outcome in Figure 4. The upper 4 panels report the results for variance estimation, while the lower four panels report results for coverage of 95% confidence intervals. Results for variance estimation were qualitatively similar to those observed in the main analysis described above (compare the upper four panels of Figure 4 with Figure 2). Similarly, results for variance estimation for the risk difference and relative work were comparable in these sensitivity as in the primary analyzes reported above (results not shown).

When estimating differences in means for continuous outcomes, empirical coverage rates of estimated 95% confidence intervals were qualitatively similar in these sensitivity to those observed in the main set of analyzes (compare the lower four panels of Figure 4 with Figure 3). Similar results were observed when estimating risk differences and relative risks (results not shown).

## 5 SECONDARY SIMULATIONS–EFFECT OF DEGREE OF OVERLAP OF PROPENSITY SCORE DISTRIBUTIONS BETWEEN GROUPS

In the extensive set of simulations described in Section 2, both the sample size and the prevalence of treatment were allowed to vary. However, the degree of overlap of the distribution of the propensity score between treated and control subjects (as measured using the c‐statistic of the propensity score model) was similar across the different scenarios. We conducted a limited set of simulations to examine the effect of the degree of overlap of the propensity score distribution between treated and control subjects on the accuracy of variance estimation. In these simulations, sample size was fixed at 1000 and the prevalence of treatment was fixed at 0.5.

### 5.1 Methods

With one modification, methods identical to those described in Sections 2.2 and 2.3 were used. The modification was in the model for simulating treatment status. The model described in Section 2.2 was modified to: $\begin{array}{l} \text{logit}\left(p_{i,\text{treat}}\right)=\alpha_{0,\text{treat}}+\sigma \log\left(1.1\right)x_{1,i}+\sigma \log\left(1.2\right)x_{2,i}+\sigma \log\left(1.5\right)x_{3,i}+\sigma \log\left(1.75\right)x_{4,i}+\sigma \log\left(2\right)x_{5,i}+\\ \;\sigma \log\left(1.25\right)x_{6,i}+\sigma \log\left(1.5\right)x_{7,i}+\sigma \log\left(2\right)x_{8,i}+\sigma \log\left(0.8\right)x_{8,i}+\sigma \log\left(0.5\right)x_{10,i}. \end{array}$

Thus, each of the regression coefficients was multiplied by a scalar σ. This would have the effect of amplifying (σ > 1) or diminishing (σ < 1) the effect of the covariates on the odds of treatment selection. We used the following nine values of σ: 0.4, 0.5, 0.6, 0.7, 0.8, 1, 1.2, 1.4, and 1.6. The distribution of the propensity score in treated and control subjects in the super‐population is described in Figure A3 in the online supplemental material. The c‐statistic of the propensity score models, which ranged from 0.64 to 0.87, are reported in each of the nine panels of Figure A3.

### 5.2 Results

**FIGURE 5:**
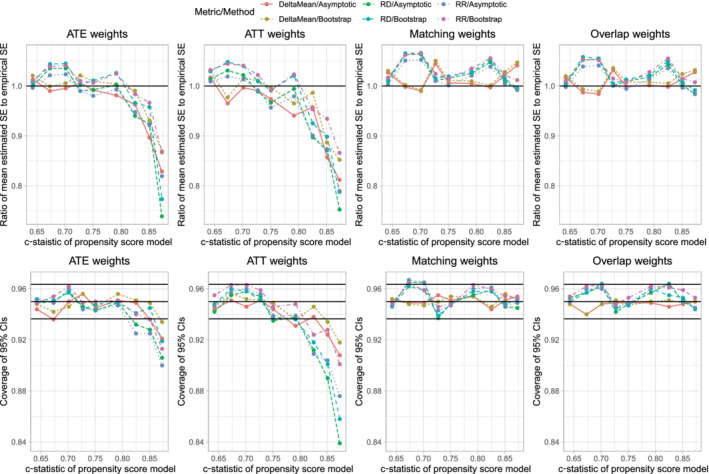
Sensitivity analysis: c‐statistic of PS model varies

Results for this secondary set of simulations are reported in Figure 5. The figure consists of eight panels. The top four panels report the ratio of the mean estimated SE to the SD of the sampling distribution of the treatment effect, while the lower four panels report the empirical coverage rates of estimated 95% confidence intervals. Note that since only one factor varied (σ), we have reported all results for all three metrics (difference in means, risk difference, and relative risk) on the same panel. When the c‐statistic of the propensity score model was high (indicating decreased overlap of the propensity score distribution between treated and control subjects), the estimated SE tended to under‐estimate the SD of the sampling distribution when using ATE and ATT weights. Variance estimation tended to be accurate when using matching weights and overlap weights. Similarly, estimated confidences when using ATE and ATT weights tended to have lower than advertised coverage when the c‐statistic of the propensity score model was high. However, estimated confidence intervals obtained using matching weights and overlap weights tended to have the advertised coverage rates, regardless of the degree of overlap of the propensity score between treated and control subjects.

## 6 SECONDARY SIMULATIONS–EFFECT OF PROPENSITY SCORE TRIMMING WITH DIFFERENT WEIGHTS

When using conventional ATE weighting, treated subjects with a low propensity score and control subjects with a high propensity score can have large weights. Large weights can result in estimated treatment effects with large variances. One approach to address this issue is to using propensity score trimming, in which subjects whose propensity score lies above or below specified thresholds are excluded from the analysis.^16^ We conducted a set of simulations to examine the effect of different trimming thresholds when using ATE weights. As in the simulations described in Section 5, sample size was fixed at 1000 and the prevalence of treatment was fixed at 0.5.

### 6.1 Methods

With one modification, the simulations were identical to those described in Section 5 that evaluated different degrees of overlap in the distribution of the propensity score. Whereas the simulations in Section 5 had one factor that was allowed to vary (σ which affects the degree of overlap of the propensity score distributions between treated and control), here we allowed two factors two vary: σ and the trimming threshold δ. The trimming threshold δ is such that subject's whose propensity score is less than δ or greater than 1−δ are excluded from the analysis. We allowed σ to take the nine values described in Section 5.1. We allowed δ to take five values: 0, 0.01, 0.02, 0.05, and 0.10 (note that δ = 0 denotes no trimming). We also considered Crump's optimal trimming threshold, which is estimated using the study sample.^16^ Once the sample had been trimmed, the propensity score was re‐estimated in the trimmed sample. We used a full factorial design with 54 scenarios (9 values of σ and 6 values of δ [including Crump's optimal threshold]).

### 6.2 Results

Across the 1000 simulation replicates for a given scenario, the median value of Crump's optimal trimming threshold ranged from 0.094 (c‐statistic of propensity score model = 0.87) to 0.131 (c‐statistic of propensity score model = 0.64). These optimal values of δ are close to Crump's pragmatic choice of 0.10.^16^

**FIGURE 6:**
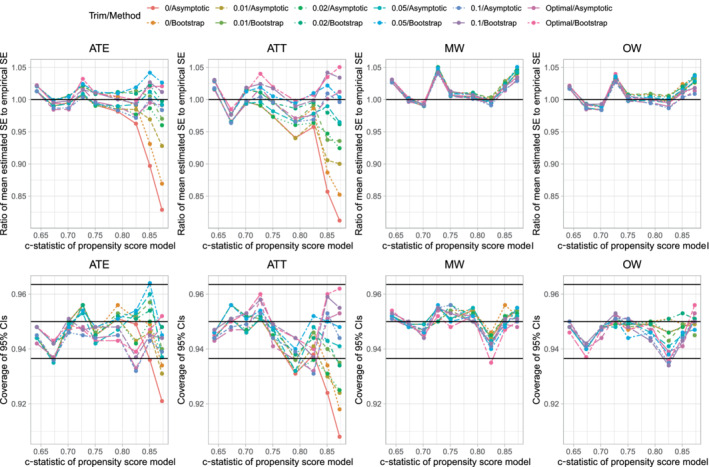
c‐statistic of PS model varies and PS trimming (difference in means)

Results are summarized in Figure 6 for the continuous outcome when the metric of treatment effect was the difference in means. The figure consists of eight panels. The upper four panels report results for the ratio of the mean estimated SE to SD of the sampling distribution, while the lower four panels report results for the empirical coverage rates of estimated 95% confidence intervals. We only report the results for estimating the difference in means of a continuous outcome. Results for estimating a risk difference and a relative risk were qualitatively similar to those for the continuous outcome.

When using ATE weights, untrimmed weights (δ = 0) resulted in estimated SEs that underestimated the SD of the sampling distribution of the difference in means when the c‐statistic of the propensity score model was ≥0.85. The use of the bootstrap improved estimation of SEs, but the standard errors were still biased low when the c‐statistic was high. When the propensity score was ≥0.85, using a trimming threshold δ ≥ 0.02 resulted in estimates of standard error with minimal bias. Qualitatively similar results were observed when using ATT weights. When using matching weights or overlap weights, the choice of trimming threshold δ had at most a negligible effect of the accuracy of the estimation of standard errors.

The use of a trimming threshold δ ≥ 0.02 when using ATE weights or δ ≥ 0.05 when using ATT weights tended to result in estimated 95% confidence intervals with the advertised coverage rate. When using matching weights or overlap weights, the choice of trimming threshold had no meaningful effect on empirical coverage rates.

## 7 CASE STUDY

We provide a case study to compare the use of the asymptotic variance estimator and the bootstrap. Our case study consists of patients discharged from hospital with a diagnosis of acute myocardial infarction (AMI or heart attack). The exposure was receipt of a prescription for a beta‐blocker at hospital discharge. Death was a binary outcome denoting death within 5 years of hospital discharge.

### 7.1 Methods

We used data, from a previously‐published study, consisting of 9107 patients who were discharged alive from hospital with a diagnosis of AMI in Ontario, Canada, between April 1, 1999 and March 31, 2001.^12^, ^17^ Baseline information was available on patient demographics, presenting signs and symptoms, classic cardiac risk factors, comorbid conditions and vascular history, vital signs on admission, and results of laboratory tests. The exposure of interest was whether the patient was prescribed a beta‐blocker at hospital discharge. Sixty‐eight percent of subjects were prescribed a beta‐blocker at hospital discharge. The outcome was a binary outcome denoting whether the patient died within 5 years of hospital discharge. Twenty‐five percent of subjects died within 5 years of hospital discharge.

We drew a random sample of size *N* from the original cohort of 9107 subjects. In this sample of size *N*, we estimated the propensity score using a logistic regression model in which treatment status was regressed on 29 baseline covariates. We then computed the four different sets of weights. We assessed the balance of baseline covariates before and after weighting using the weighted standardized difference^12^ and reported the maximum absolute standardized difference across the 29 baseline covariates. We also assessed the degree of overlap of the propensity score distribution between treated and control subjects, before and after weighting, using the overlapping coefficient.^18^ The overlapping coefficient, which is the complement of the proportion of overlap between two density functions, ranges from 0 to 1, with a value of 1 denoting no overlap and a value of zero denoting perfect overlap. We then estimated the risk difference using each set of weights. Standard errors were estimated both using the asymptotic variance estimator and using 200 bootstrap samples. Ninety‐five percent confidence intervals were constructed using normal‐theory methods. This process was repeated allowing *N* to range from 500 to 9000 in increments of 500.

### 7.2 Results

**FIGURE 7:**
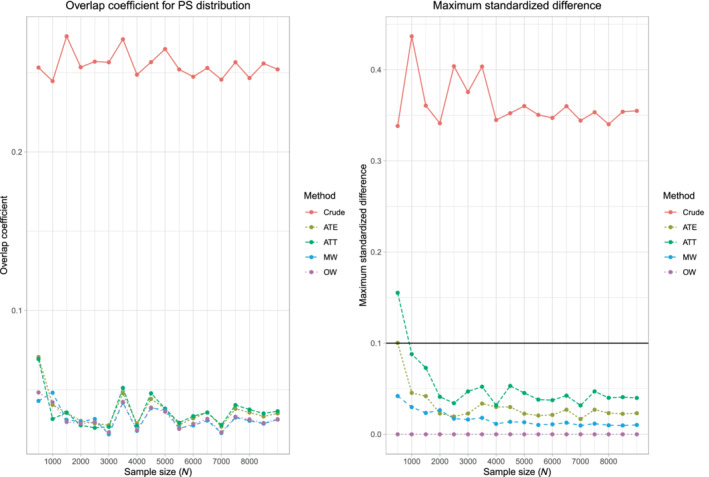
Case study—overlap and balance diagnostics

**FIGURE 8:**
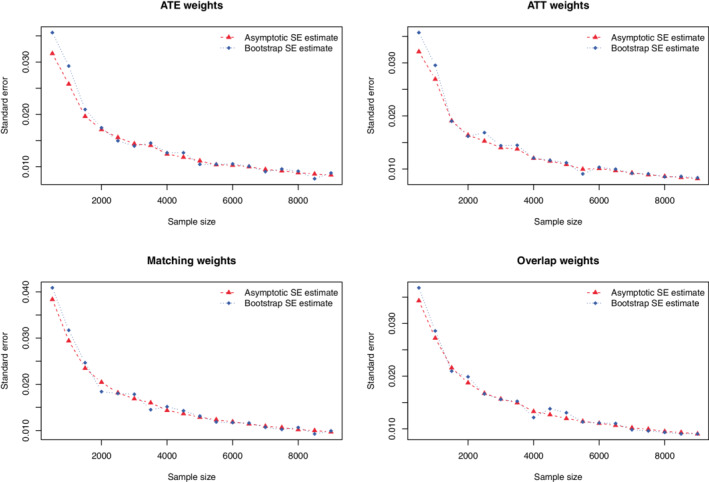
Case study—comparison of estimated standard errors

**FIGURE 9:**
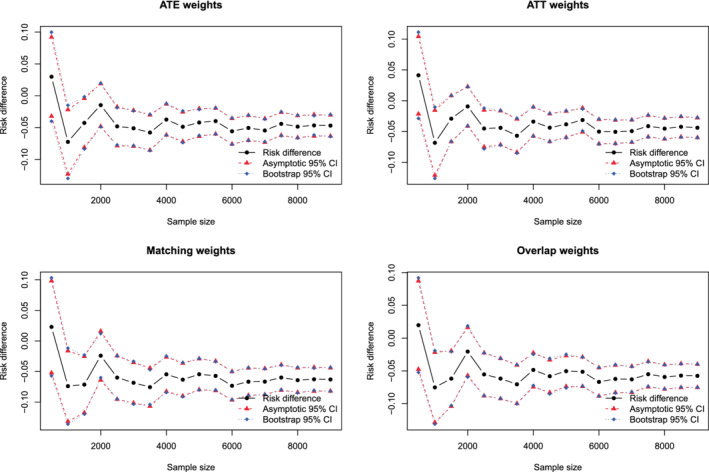
Case study—comparison of 95% confidence intervals

Results are summarized in Figure 7 (overlap and balance), Figure 8 (estimated SEs) and Figure 9 (estimated 95% confidence intervals). The value of the overlapping coefficient is reported in the left panel of Figure 7. Incorporating each set of weights improved the degree of overlap of the propensity score distribution between treated and control subjects compared to the unweighted sample. There were no meaningful differences in the overlapping coefficient between the four different sets of weights. In the unweighted sample, the maximum standardized difference exceeded 0.30 across all values of *N*. With one exception, when incorporating the weights, the maximum standardized difference was less than 0.10 across all values of *N*. The maximum standardized difference was largest when using the ATT weights and smallest when using overlap weights.

When using ATE weights, the asymptotic estimate of the SE of the risk difference agreed with the bootstrap estimate once the sample size was at least 2000. When the sample size was less than 2000, the bootstrap estimate of the SE was larger than the asymptotic estimate. When using ATT weights, the asymptotic estimate of the SE of the risk difference agreed with the bootstrap estimate once the sample size was at least 1000. When the sample size was less than 1000, the bootstrap estimate was larger than the asymptotic estimate. When using matching weights or overlap weights, the two estimates of the SE were very similar across all sample sizes. Results on the similarity of estimated confidence intervals reflect those observed for constructed 95% confidence intervals (which was to be expected as we used normal‐theory methods to construct the confidence intervals).

## 8 DISCUSSION

We compared the performance of asymptotic variance estimators with that of the bootstrap when estimating SEs and constructing confidence intervals when using propensity score‐based weighting. We considered four different sets of weights: ATE weights, ATT weights, matching weights, and overlap weights.

We summarize our findings as follows: first, when using ATE weights and sample sizes were ≤ 1000, then the use of the bootstrap resulted in estimates of SE that were more accurate than the asymptotic variance estimates. The magnitude of inaccuracy increased as the prevalence of treatment moved away from 0.5. We hypothesize that, when applying this finding to empirical studies in different settings, that similar results would be expected to be true, with the magnitude of bias depending on study characteristics. Second, when using ATT weights and sample sizes were small to moderate and the prevalence of treatment was moderate to high, then the use of the asymptotic variance estimator resulted in estimates that were less accurate than those obtained using the bootstrap. Third, when using matching weights and overlap weights, both the asymptotic estimator and the bootstrap resulted in accurate estimates of SE across all sample sizes and prevalences of treatment. Fourth, even when using the bootstrap with ATE weights, empirical coverage rates of confidence intervals were suboptimal when sample sizes were low to moderate and the prevalence of treatment was either very low or very high. Fifth, even when using the bootstrap with ATT weights, empirical coverage rates of confidence intervals were suboptimal when sample sizes were low to moderate and the prevalence of treatment was very high. We would note that, in general, one would often use ATT weights in settings in which the prevalence of treatment was low to moderate. Sixth, when using matching weights and overlap weights, both the asymptotic estimator and the bootstrap resulted in confidence intervals whose empirical coverage rates tended to not differ from the advertised rate.

The asymptotic variance estimators used in the current study account for the uncertainty in estimating the propensity score model.^14^ Had the asymptotic variance estimator not accounted for uncertainty in the estimate propensity score, one would anticipate that the estimated SEs would be too small, as all of the sources of variation had not been accounted for. The bootstrap estimator implicitly accounts of the uncertainty in estimating the propensity score model, as the propensity score model is re‐estimated in each bootstrap sample. Thus, the differences observed between the accuracy of the asymptotic estimates and that of the bootstrap estimates when sample sizes were small is not simply because one method accounts for uncertainty in the estimated propensity score and the other did not. Our findings suggest that, as would be expected, the accuracy of the closed‐form asymptotic estimators improves as sample size increases.

The comparison of asymptotic estimates of standard errors with those obtained using the bootstrap may seem irrelevant to applied research, as there is a perception that propensity score methods are used exclusively with very large samples. However, this perception is incorrect. Sturmer et al reviewed 177 studies published in the medical literature that used propensity score methods.^19^ Of the 176 studies that reported sample sizes, the 25th percentile of sample size was 687, while 33% of studies had sample sizes less than 1000. Thus, a sizeable minority of studies that used propensity score methods were conducted using sample sizes in which the bootstrap estimate of SE would be preferable to asymptotic estimates when estimating the effect of treatment on continuous or binary outcomes.

There is a limited literature on the use of the bootstrap with propensity score weighting. Austin showed that a robust (or sandwich) variance estimator had worse performance than that of the bootstrap when estimating marginal hazards ratios using a Cox proportional hazard model with either ATE or ATT weights,^20^ with the robust variance estimator tending to overestimate the sampling variability of the log‐hazard ratio. Mao et al examined variance estimation of the log‐hazard ratio when using ATE weights, matching weights and overlap weights in settings with sample sizes of 500 or 1000.^21^ When using ATE weights and they found that the robust variance estimator for the log‐hazard ratio overestimated the sampling variability of the marginal log‐hazard ratio when there was good overlap between groups and underestimated the sampling variability when there was poor overlap between groups and sample size was 500. In contrast to this, the use of the bootstrap resulted in approximately correct variance estimates when there was good overlap between groups and underestimated the sampling variability when there was poor overlap between groups. When using matching weights or overlap weights, the robust variance estimator resulted in overestimates of the sampling variability, regardless of whether there was good or poor overlap between groups, while the bootstrap resulted in correct estimates of sampling variability. Raad et al examined the use of IPTW to adjust for baseline covariates in small randomized controlled trials.^22^ They found that, when the sample size was between 100 and 150, the coverage of confidence intervals derived using the asymptotic variance estimator was slightly below the advertised rate, while the coverage rate was significantly below the advertised rate when the sample size was less than 100. When adjusting for 1 or 2 covariates, the bootstrap estimate of SE performed similarly to the asymptotic variance estimator as the sample size ranged from 40 to 200. However, the bootstrap estimator had worse performance than the asymptotic estimator when adjusting for 4 covariates (and sample size was less than 80) and when adjusting for 6 covariates (and sample size was less than 110).

The current study was restricted to settings with binary treatments (eg, active treatment vs control). Yoshida et al extended matching weights for use in settings with three treatment groups.^23^ Similarly, Li and Li developed a class of balancing weights for use in settings with multiple treatments.^13^ Further research is required to extend our evaluation to settings with nonbinary treatments. As noted by Rubin, one option when considering categorical (nonbinary) exposures is that one can conduct a sequence of pair‐wise comparisons of treatments.^24^ When using this approach, the findings of our study would be directly applicable to these pair‐wise comparisons.

In the current study we focused on treatment effect estimation when outcomes were continuous or binary. Survival or time‐to‐event outcomes are also of interest in medical and epidemiologic settings.^25^ Mao et al describe estimators, for both the point estimate and for the SE, when using propensity score‐based weighting to estimate marginal hazard ratios with survival outcomes.^21^ As noted above, an earlier study examined the use of the bootstrap when using propensity score‐based weighting to estimate hazard ratios.^20^ Zeng et al developed a method based on pseudo‐observations for the analysis of survival outcomes when using propensity score weighting.^26^ Cheng et al developed estimators for survival functions when using propensity score‐based weights.^27^ In the current study we focused on estimating the effect of treatment in the overall sample. Yang et al extended the use of overlap weights for use when estimating subgroup‐specific treatment effects.^28^ The proposed method allowed for achieving exact balance within subgroups. The current study was focused on the use of different sets of weights to estimate the effect of treatment in observational (or nonrandomized) studies. Both Zeng et al and Yang et al described the use of overlap weights in randomized controlled trials.^29^, ^30^ The use of weighting allows the analyst to reduce the effects of residual imbalance in measured baseline covariates between treatment groups.

There are certain limitations to the current study. The primary limitation of the study is that it is based on Monte Carlo simulations. Such an approach is necessary due to our studying the performance of the bootstrap in the context of propensity score weighting, a setting in which analytic calculations are not feasible. However, we increased the utility of our simulations by considering settings with small, medium, and large sample sizes. Furthermore, we allowed the prevalence of treatment to range from very low (10%) to very high (90%). Furthermore, we included an extensive set of sensitivity analyzes to examine different settings. A second limitation is that we restricted our examination to one bootstrap method for constructing confidence intervals. We used normal‐theory methods using the bootstrap estimate of the SE to construct confidence intervals. We did not examine the performance of percentile‐based bootstrap confidence intervals. This decision was made primarily due to the increased computational demands required when constructing percentile‐based bootstrap confidence intervals. While the bootstrap estimate of the SE can be obtained using 200 bootstrap replicates, it has been suggested that 2000 bootstrap replicates be used when constructing percentile‐based confidence intervals. The current simulations required approximately 2 weeks of CPU time when using 200 bootstrap replicates. Using 2000 bootstrap replicates to construct percentile‐based bootstrap confidence intervals would have required approximately 20 weeks of CPU time. A third limitation was the restriction to settings with binary exposures and to outcomes that were either continuous or binary. As noted above, weighting‐based methods have been extended for use with multiple treatments and with survival outcomes.

We evaluated the performance of variance estimators when using four different sets of weights based on the propensity score. It is important to emphasize that the applied analyst should not necessarily select the weights with the best performing variance estimator. Each set of weights has a different target estimands. ATE weights allow for estimation of the ATE: the effect of moving the entire population (sample) from control to treated. ATT weights allow for estimation of the ATT: the effect of treatment in those subjects who were ultimately treated. The use of matching weights and overlap weights focus on estimating the effect of treatment in those subjects for whom there is clinical equipoise.^4^ The choice of weights should be motivated, at least in part, by which set of weights allows the investigator to address the specific study question. Furthermore, the population to whom the ATE pertains can be easily *defined* using the study inclusion and exclusion criteria. However, it is more difficult to formally *define* the population in which there is clinical equipoise. While this population can be *described* using descriptive statistics, it is more difficult to formally *define* this population.

We provide the following recommendations: first, when using matching weights or overlap weights, the asymptotic variance estimator can be used with sample sizes as small as 250 (as long as the prevalence of treatment is not less than 0.1 and not greater than 0.9, which were the smallest and highest prevalences that we considered). Second, when using ATE weights, one should use the bootstrap estimator when the sample size is 1000 or less. Third, when using ATT weights, one should use the bootstrap estimator if the sample size is 1000 or less and the prevalence of treatment is at least moderately high. Fourth, researchers should be cognizant that, even with the use of the bootstrap, constructed confidence intervals may have suboptimal coverage rates when sample sizes are low to moderate and the prevalence of treatment is either very low or very high (ATE weights) or very high (ATT weights).

## Supporting information


**Figure A1** Overlap in propensity score distribution between treated and control subjects
Figure A2. Effective sample size
Figure A3. Overlap in propensity score distribution between treated and control subjects for different scalars

## Data Availability

The dataset from this study is held securely in coded form at ICES. While legal data sharing agreements between ICES and data providers (e.g., healthcare organizations and government) prohibit ICES from making the dataset publicly available, access may be granted to those who meet prespecified criteria for confidential access, available at www.ices.on.ca/DAS (email: das@ices.on.ca).
