# Supplementary material for: Bootstrap vs asymptotic variance estimation when using propensity score weighting with continuous and binary outcomes
Source: Stat Med. 2022 Jul 15;41(22):4426–43. doi: 10.1002/sim.9519 (PMC9544125; doi:10.1002/sim.9519)

Figure A1. Overlap in propensity score distribution between treated and control subjects

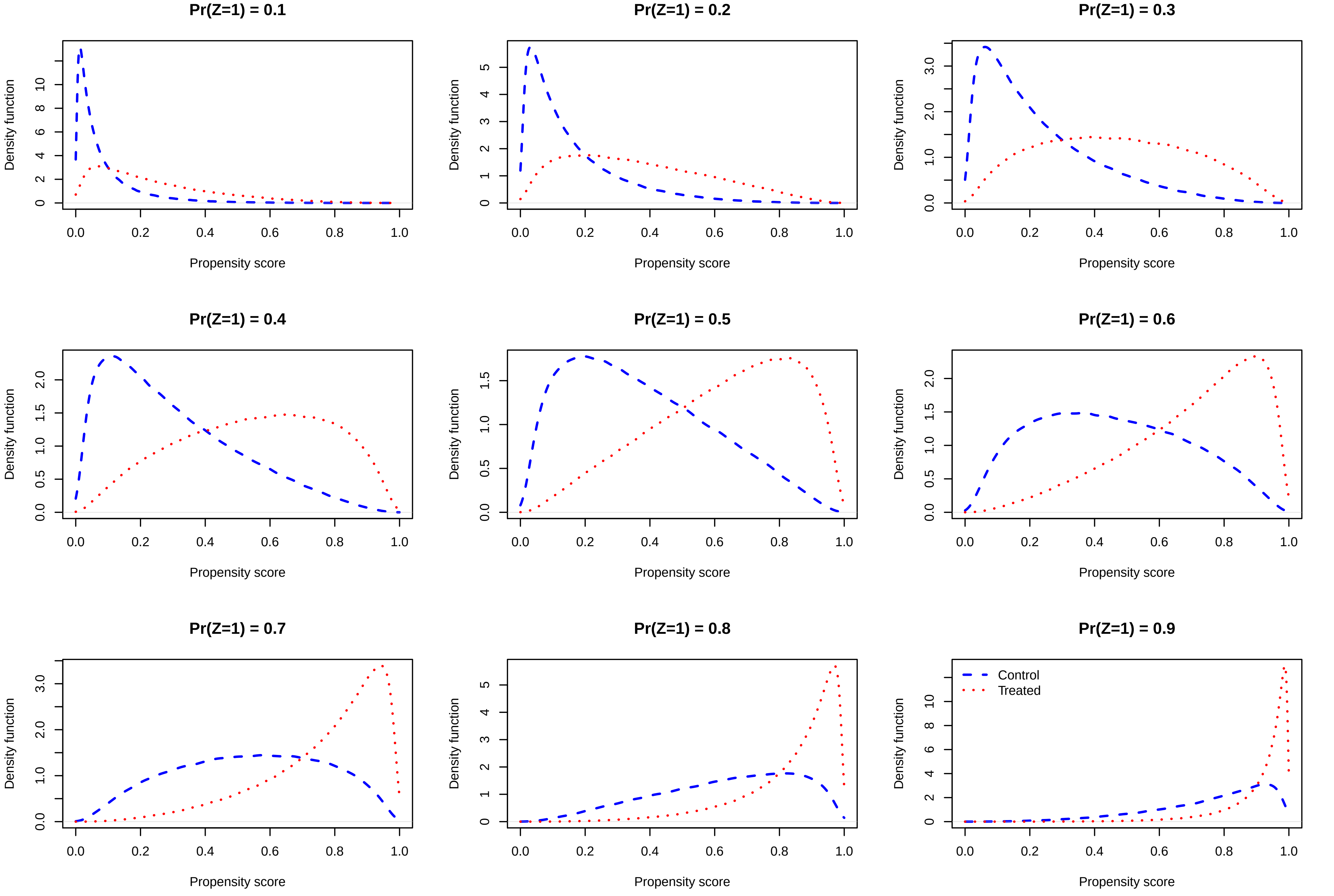

Figure A2. Effective sample size

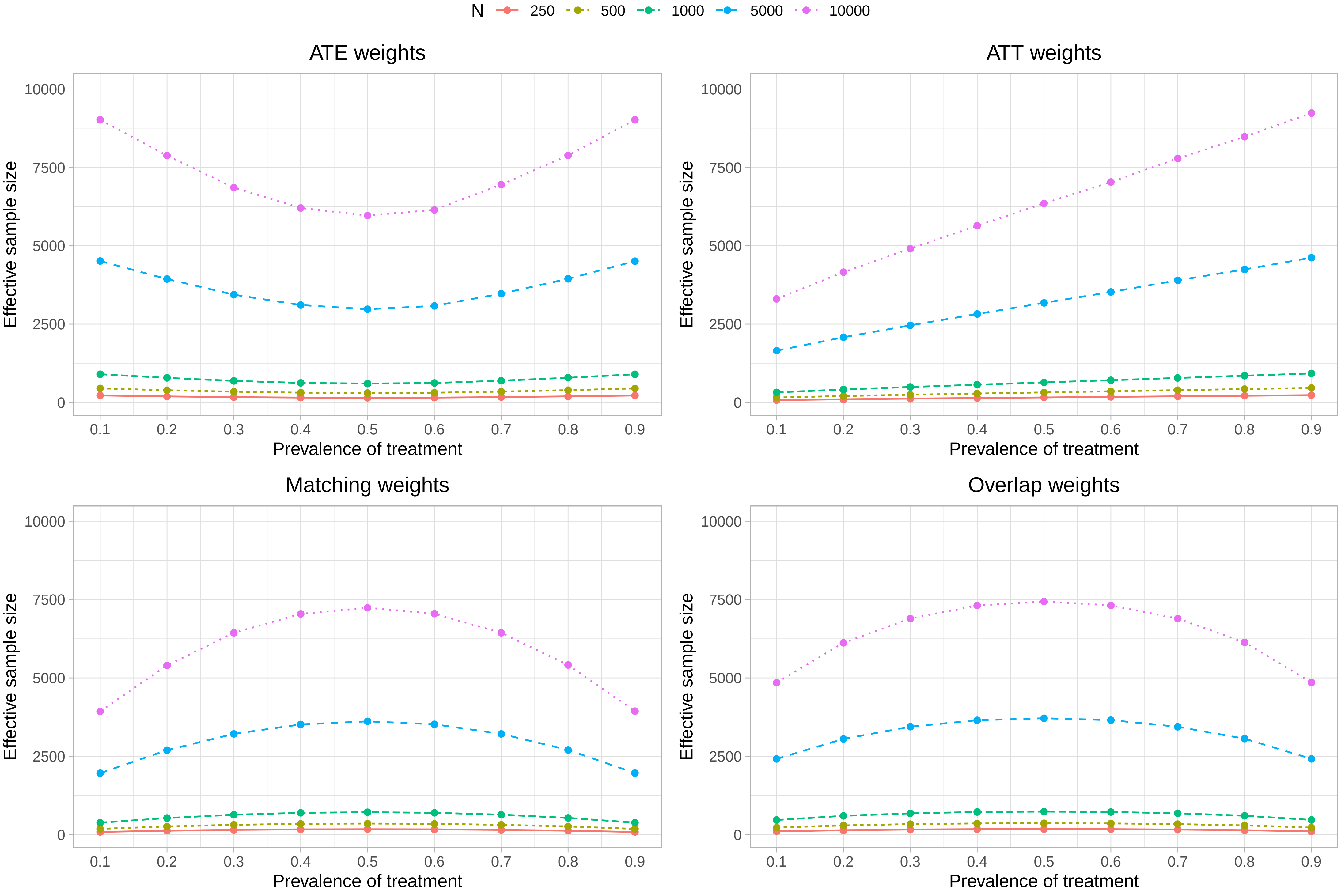

Figure A3. Overlap in propensity score distribution between treated and control subjects for different scalars

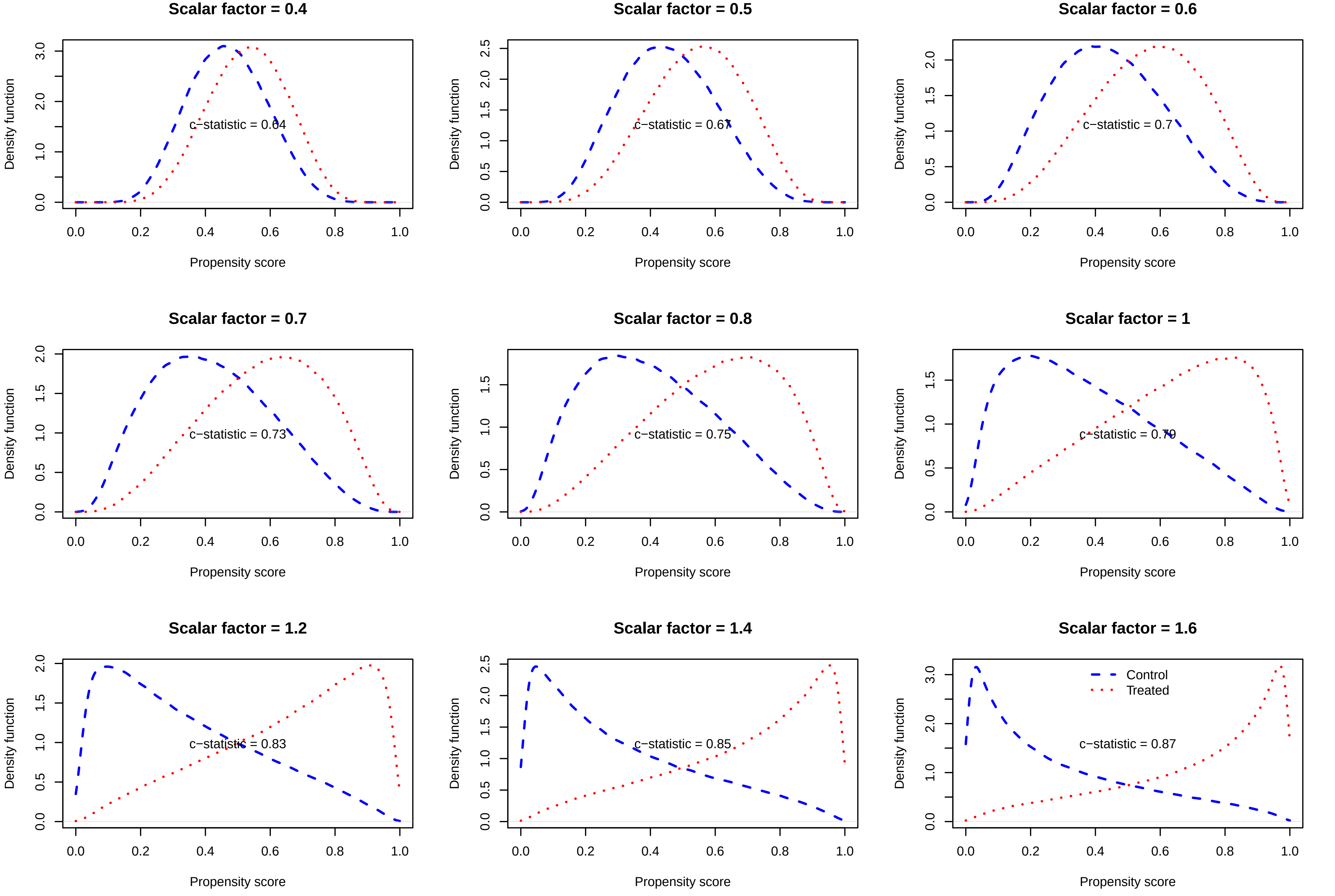

Supplement: Supplementary file 1 — Figure A1 Overlap in propensity score distribution between treated and control subjects Figure A2. Effective sample size Figure A3. Overlap in propensity score distribution between treated and control subjects for different scalars [file SIM-41-4426-s001.pdf]
